# Correlation between altered gut microbiota and elevated inflammation markers in patients with Crohn’s disease

**DOI:** 10.3389/fimmu.2022.947313

**Published:** 2022-08-15

**Authors:** Jun Hu, Sijing Cheng, Jiayin Yao, Xutao Lin, Yichen Li, Wenxia Wang, Jingrong Weng, Yifeng Zou, Lixin Zhu, Min Zhi

**Affiliations:** ^1^ Department of Gastroenterology, The Sixth Affiliated Hospital of Sun Yat-sen University, Guangzhou, China; ^2^ Guangdong Institute of Gastroenterology, Guangzhou, China; ^3^ Guangdong Provincial Key Laboratory of Colorectal and Pelvic Floor Disease, The Sixth Affiliated Hospital, Sun Yat-sen University, Guangzhou, China; ^4^ Department of Colorectal Surgery, The Sixth Affiliated Hospital, Sun Yat-sen University, Guangzhou, China; ^5^ School of Medicine, Sun Yat-sen University, Shenzhen, China

**Keywords:** gut microbiome, inflammatory bowel disease, short chain fatty acid, multivariate regression, metabolomics

## Abstract

Prior studies reported inconsistent results on the altered gut microbial composition in patients with Crohn’s disease (CD), likely under the influences of many confounding factors including genetic, life style and environmental variations among different study cohorts. This study aims to examine the gut microbiota of CD patients with particular efforts to minimize the impact of the confounding factors. For this purpose, the healthy relatives of the patients were enrolled as control subjects so that the paired study subjects may have similar genetic background, dietary habits, and household environment. The fecal microbiota of the study subjects were examined by 16S rRNA sequencing. After the identification of the differential bacterial genera, multivariate regression analysis was performed to adjust the results for the impact of confounding factors. We found that the microbiota of the CD patients were featured with reduced short chain fatty acid (SCFA) producing bacteria and elevated opportunistic pathogen *Escherichia-Shigella*. Correlation analysis indicated that the elevation in *Escherichia-Shigella* and the reduction in SCFA-producing bacteria usually occur simultaneously. These differential genera exhibited a high capacity in distinguishing between CD and healthy controls achieving an area under curve of 0.89, and were correlated with the changes in inflammation related blood biochemical markers. Consistent with the reduction in SCFA-producing bacteria in CD, metabolomics analysis revealed decreased blood level of SCFAs in the patients. The differential genera identified in this study demonstrated outstanding capability to serve as diagnosis markers for CD and are potential targets for intervention.

## Introduction

Crohn’s disease (CD) is an inflammatory bowel disease characterized by chronic inflammation of any part of the gastrointestinal tract. CD has a progressive and destructive course and is increasing in incidence worldwide. The etiology of CD is not fully understood but it is thought to develop as a result of interactions between environmental, microbial, and immune-mediated factors in a genetically susceptible host (1). Gut dysbiosis has been observed in many gastrointestinal diseases (2–5), including CD. An imbalance of microbiota may be an important indicator of the occurrence and development of CD (6).

Millions of microbes are known to constantly interact with the host within the human system such as the gastrointestinal tract (7). The gut microbiota includes opportunistic pathogens and non-opportunistic pathogens. These pathogens and their fermentation products may translocate from the gut lumen to other tissues and organs, when the gut mucosal barrier is impaired or when antibiotics or nutrition deficiency destroys the balance of the gut community (8). The gut microbiota is deemed critical in stimulating the immune response and preventing pathogenic infections of the host (9). Meanwhile, the metabolic activity of microbiome is also important for host health, and dietary fiber is used as a source of energy by microbiome. Carbohydrates resulting from the degradation of polysaccharides in fiber are fermented into short chain fatty acids (SCFAs) such as acetate, propionate, and butyrate. The SCFAs are a fundamental source of energy for intestinal epithelial cells, have a gut barrier function and important immunomodulatory functions. Bacteria that ferment fiber and produce SCFAs (including Roseburia, Faecalibacterium, Prevotella9 and Coprococcus) have been reported to be significantly reduced in abundance in the mucosa and feces of patients with CD (10, 11).

However, other studies did not observe reduced abundance in SCFA-producing bacteria in the microbiota of patients with CD (12, 13). Instead, these authors reported increased abundances in Enterobacteriaceae, Pasteurellacaea, Veillonellaceae, and Fusobacteriaceae, and decreased abundance in Erysipelotrichales, Bacteroidales and Clostridiales in the gut microbiota of CD patients (13). The inconsistencies could be the consequences of many confounding factors including genetic variations within and between the study cohorts, different life styles such as diet and personal hygiene, and different environment between the study cohorts. Therefore, to better understand the dysbiosis in the gut of CD, the genetic, lifestyle and environmental variations need to be considered in the microbiome study. To this end, we examined the microbiome and metabolome of the CD patients, in comparison to their healthy relatives. We identified 10 abundant differential genera including *Escherichia-Shigella*, *Atlantibacter*, and SCFA-producing genera between CD and controls. Some of these genera were correlated with the changes in inflammation related blood biochemical markers. Functional analysis indicated that altered gut microbiome may contribute to CD pathogenesis *via* two mechanisms.

## Results

### Participant characteristics

A total of 91 CD patients and 91 paired control subjects (their healthy relatives) were included in our study. The major demographic and baseline clinical features of the study groups are presented in **Table 1**. The body mass index (BMI) at diagnosis was lower in the CD patients compared to their healthy relatives (P < 0.0001). There was no significant difference between the CD patients and their healthy relatives with respect to gender and age. Many CD patients exhibited elevated levels of erythrocyte sedimentation rate (ESR), C-reactive protein (CRP), and platelet count, in consistent with the ongoing intestinal inflammation. On the other hand, many CD patients had decreased levels of albumin and hemoglobin, reflecting compromised nutrition absorption in the intestines. Ileocolonic inflammation were reported for the majority of the CD patients. Treatments to control intestinal inflammation included aminosalicylic acid, corticosteroids, immunomodulators, and biologics.

**Table 1:** Characteristics of the participants.

	Reference values	CD (n=91)	Control (n=91)	*P* value^a^
Age (years)		28.84 ± 7.24^b^	28.6 ± 7.38	0.822
Gender (male/female)		61/30	52/39	0.169
BMI (kg/m^2^)	18.5-23.9	18.64 ± 2.91	21.5 ± 3.56	<0.001
White blood cell count (x10^9^/L)	4.00-10.00	7.19 ± 3.17	NA	
Platelet count (x10^9^/L)	100.00-300.00	326.9 ± 136.10	NA	
ESR (mm/1h)	0.00-20.00	36.59 ± 27.59	NA	
CRP (mg/L)	0.00-10.00	34.53 ± 37.24	NA	
ALB	40.00-55.00	42.40 ± 30.38	NA	
Hb	120.00-160.00	114.80 ± 24.40	NA	
Disease location				
L1 ileal (%)		18 (19.78%)	NA	
L2 colonic (%)		7 (7.69%)	NA	
L3 ileocolonic (%)		59 (64.84%)	NA	
L1+L4 ileal+isolated upper disease (%)		4 (4.40%)	NA	
L3+L4 ileocolonic+isolated upper disease (%)		3 (3.30%)	NA	
Treatment				
Aminosalicylic acid (%)		29 (31.87%)	NA	
Corticosteroids (%)		14 (15.38)	NA	
Immunomodulators (%)		34 (37.36%)	NA	
Biologics (%)		14 (15.38%)	NA	
Antibiotics		0	NA	

ALB, Albumin; BMI, Body mass index; CRP, C-reactive protein; ESR, Erythrocyte sedimentation rate; Hb, Hemoglobin; NA, Not available; ^a^P values are from paired t test, Chi-square test, as appropriate; ^b^mean ± standard error of the mean.

### Altered ecological diversity in the gut of CD patients compared to their healthy relatives

The gut microbiome profiles of the CD patients and controls were analyzed using 16S rRNA gene sequencing method. After removing the low quality reads, a total number of 3,161,793 sequencing reads were obtained from the 182 samples, with an average number of 15,950 reads (minimum length 6,290nt, maximum length 50,912nt).

**Figure 1:**
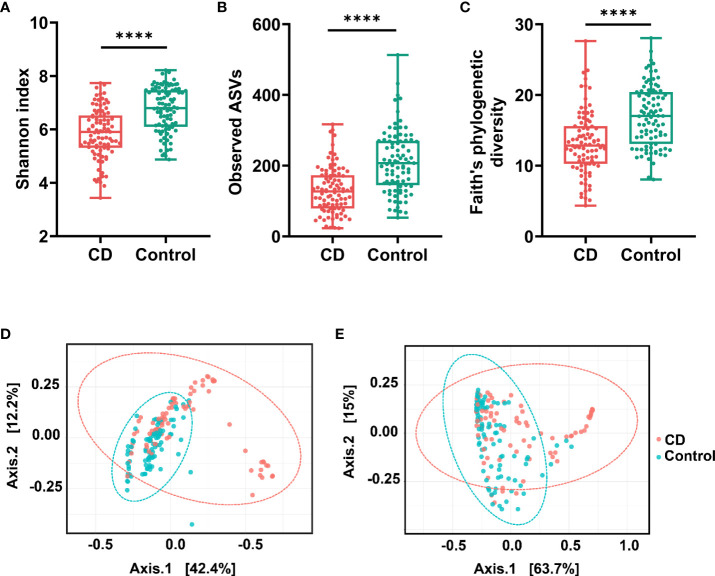
α diversities and β diversities in the gut of Crohn’s disease (CD) and control groups. Box plots are microbial α diversities in CD and controls. The top and the bottom whiskers indicate the maximum and the minimum values, respectively, and the hyphen represents the median value. The differences of α diversities, including Shannon **(A)**, observed ASVs **(B)**, and Faith’s phylogenetic diversity **(C)** at the ASV level were evaluated by paired t-test. β diversities calculated by UniFrac based unweighted **(D)** and weighted **(E)** principal coordinate analysis (PCoA). Permutational multivariate analysis of variance (PERMANOVA) was conducted to assess the difference of beta diversity between CD and control group. All comparisons were significantly different with *P* < 0.0001. #**** indicates p < 0.0001.

The variations of microbial communities within sample (α diversity) and between samples (β diversity) in CD patients and their healthy relatives were estimated at the amplicon sequence variant (ASV) level. Based on the metrics Shannon index, observed ASVs, and Faith’s phylogenetic diversity, the α diversity in the gut was significantly decreased in CD compared to the controls (**Figures 1A**−**C**). For β diversities, the principal coordinate analysis (PCoA) based on unweighted and weighted UniFrac distance was conducted and plotted to compare the microbial communities between CD patients and their healthy relatives. Significant differences between the two groups were observed with both unweighted and weighted analyses (PERMANOVA test, P < 0.0001; **Figures 1D, E**).

### Abnormal gut microbiota composition in CD patients compared to their healthy relatives

**Figure 2:**
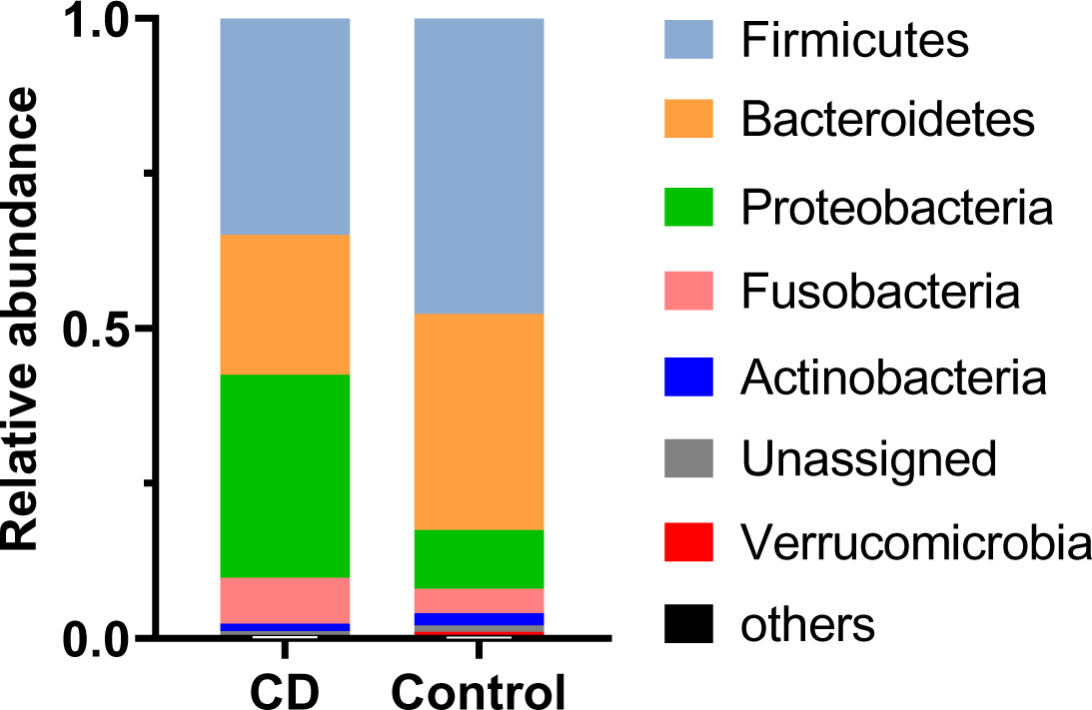
Differential phylum distribution of the gut microbiome in CD and control groups. Average relative abundances of individual phylum are plotted.

Abundant differences in microbial compositions were observed between CD patients and their healthy relatives at multiple taxonomic levels. Among 19 bacterial phyla identified in this study, the top four abundant phyla were Firmicutes, Bacteroidetes, Proteobacteria and Fusobacteria, together accounting for 97.60% and 95.91% average sequencing reads in the CD and control groups, respectively (**Table 2**, **Figure 2**). Both Firmicutes and Bacteroidetes showed decreased abundances in CD patients compared to healthy controls, while Proteobacteria and Fusobacteria exhibited increased abundances in CD patients compared to their healthy relatives (**Table 2**, **Figure 2A**).

**Table 2:** Abundant taxa in the gut microbiota of IBD patients and their healthy relatives.

Phylum	Family	Genus	CD (n=91)	Control (n=91)	*P* value^a^
**Firmicutes^b^**			**34.91^c^**	**47.66**	**<0.001**
	**Lachnospiraceae^d^**		**13.41**	**22.84**	**<0.001**
		Blautia^e^	2.09	3.11	0.141
		Anaerostipes	0.67	1.19	0.129
		**Roseburia**	**0.34**	**1.56**	**<0.001**
		**Agathobacter**	**0.21**	**1.04**	**0.001**
		**Lachnospira**	**0.15**	**1.06**	**<0.001**
		[Ruminococcus] gnavus group	2.43	1.40	0.108
		Lachnoclostridium	1.85	1.47	0.203
	Veillonellaceae		7.51	5.79	0.352
		**Veillonella**	**4.04**	**0.32**	**0.003**
		Megamonas	2.30	4.26	0.139
	**Ruminococcaceae**		**3.51**	**10.05**	**<0.001**
		**Faecalibacterium**	**1.45**	**3.81**	**<0.001**
	Acidaminococcaceae		2.64	4.07	0.121
		Phascolarctobacterium	2.45	3.85	0.122
	Peptostreptococcaceae		2.11	1.63	0.539
		**Romboutsia**	**0.50**	**1.37**	**0.002**
	Lactobacillaceae		1.11	0.08	0.139
		Lactobacillus	1.10	0.08	0.145
**Bacteroidetes**			**22.56c**	**34.85**	**<0.001**
	**Prevotellaceae**		**1.57**	**11.72**	**<0.001**
		**Prevotella9**	**1.41**	**10.96**	**<0.001**
	Rikenellaceae		0.94	1.74	0.085
		Alistipes	0.93	1.71	0.094
	Bacteroidaceae		17.30	17.30	0.997
		Bacteroides	17.30	17.30	0.997
	Tannerellaceae		2.20	2.54	0.619
		Parabacteroides	2.14	2.51	0.597
**Proteobacteria**			**32.71**	**9.45**	**<0.001**
	**Enterobacteriaceae**		**29.95**	**5.08**	**<0.001**
		**Escherichia-Shigella**	**13.92**	**1.76**	**<0.001**
		**Atlantibacter**	**13.57**	**2.35**	**<0.001**
		Klebsiella	1.98	0.89	0.285
	Burkholderiaceae		1.77	3.06	0.107
		Sutterella	1.01	0.92	0.807
		Parasutterella	0.72	1.99	0.07
Fusobacteria			7.42	3.94	0.072
	**Fusobacteriaceae**		**7.41**	**3.94**	**0.047**
		**Fusobacterium**	**7.39**	**3.94**	**0.048**
Actinobacteria			1.23	1.98	0.12
	Bifidobacteriaceae		0.81	1.27	0.105
		Bifidobacterium	0.80	1.27	0.101

^a^p values are from paired t test. ^b^Phyla with average abundance greater than 1% in any of the groups are listed. ^c^Numbers listed under study groups are percentages. ^d^Families with average abundance greater than 1% in any of the groups are listed. ^e^Genera with average abundance greater than 1% in any of the groups are listed.

Bold indicates statistical significance.

**Figure 3:**
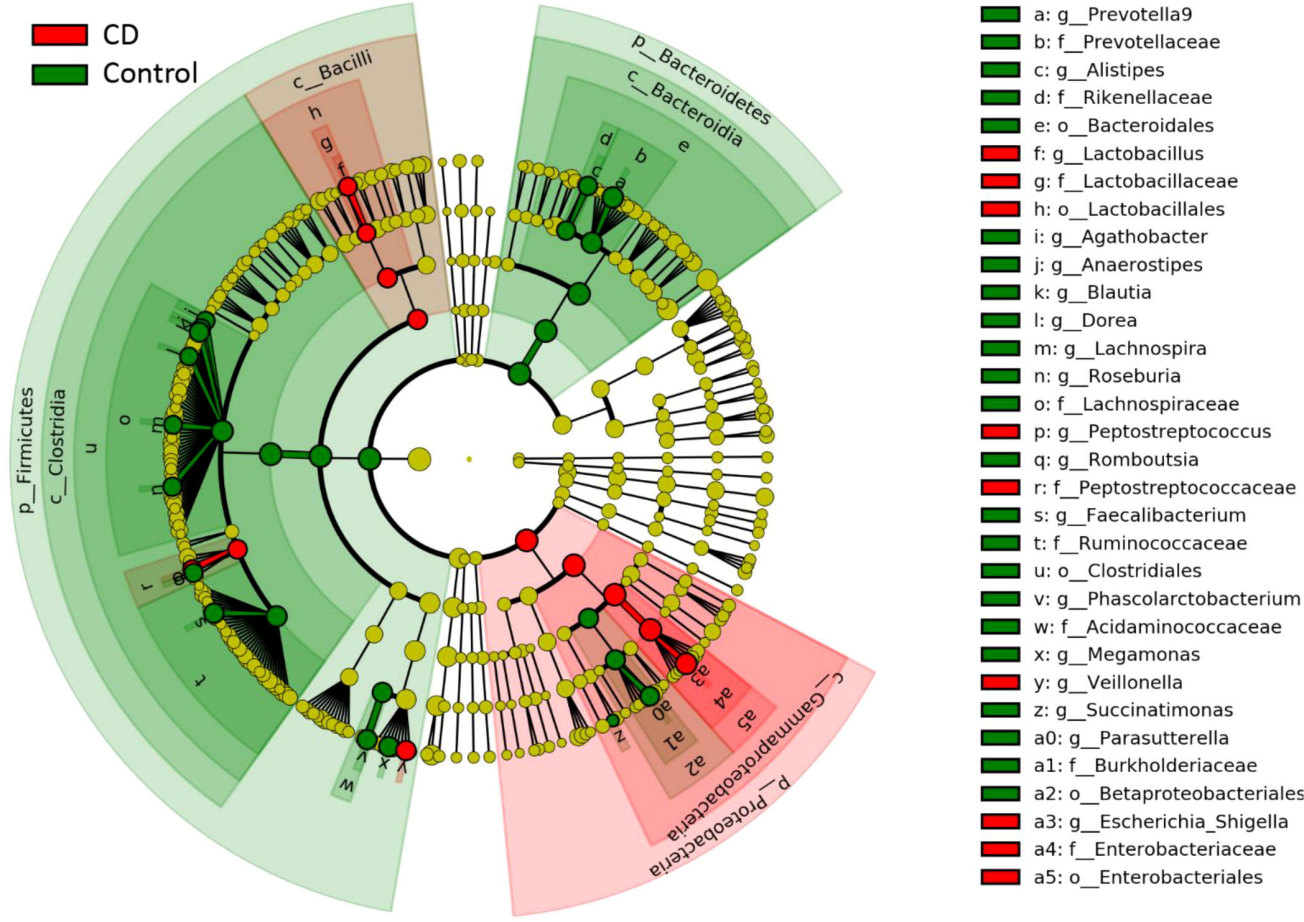
Differential gut bacterial taxa between CD and control groups. Cladogram of LEfSe linear discriminant analysis of the microbial composition comparing CD patients and controls with 16S rDNA sequencing data. Red and green indicate taxa enriched in CD or control group, respectively. The diameter of each circle is proportional to the relative abundance of the taxon.

**Figure 4:**
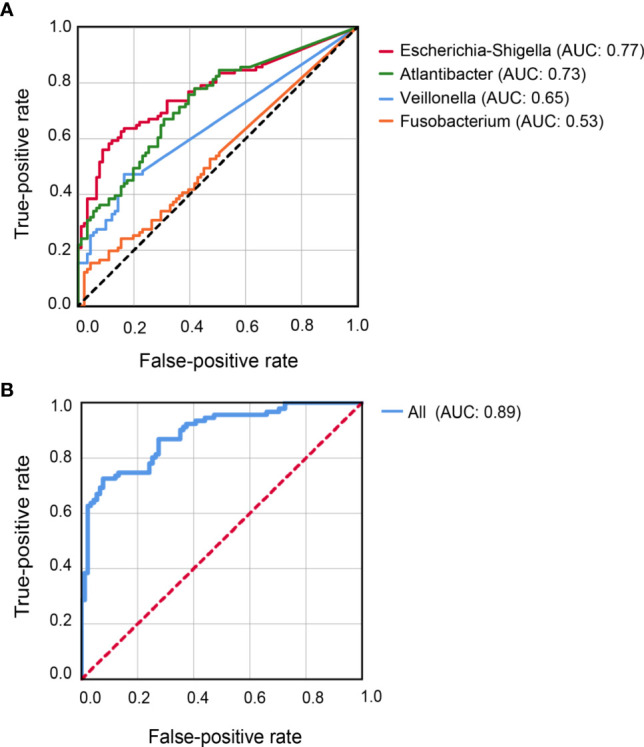
Receiver-Operator Curve plots of microbial markers for distinguishing CD patients from healthy controls. **(A)** Models with individual genus marker. **(B)** Model with combined genus marker. AUC, area under the curve.

Differential taxa at every taxonomic levels were identified using the Linear discriminant analysis Effect Size (LEfSe) method with the threshold of a linear discriminant analysis (LDA) score at 3.5. At the level of genus, 18 differential genera between the CD patients and their healthy relatives were identified (**Figure 3**). Excluding those with a microbiota abundance less than 1%, 10 genera were identified as potential disease markers for CD (**Figure 4A**). Among them, *Roseburia, Agathobacter, Lachnospira, Faecalibacterium, Romboutsia* and *Prevotella 9* exhibited lower abundances in CD; whereas *Veillonella, Fusobacterium, Escherichia-Shigella* and *Atlantibacter* were elevated in the CD group compared to the control group (**Figure 4A**).

To ascertain the differential representation of the above genera between the study groups, logistic regression was performed these 10 abundant differential genera. Univariate analysis revealed that, lower abundances of *Prevotella 9, Agathobacter, Lachnospira, Roseburia, Romboutsia, Faecalibacterium* and higher abundances of *Veillonella, Atlantibacter, Escherichia-Shigella* were significantly associated with CD. After adjusting for the influences of age and gender, multivariate analyses arrived at the same result, that is, lower abundances of *Prevotella 9, Acanthocytes, Lachnospira, Roseburia, Romboutsia, Faecalibacterium* and higher abundances of *Veillonella, Atlantibacter, Escherichia-Shigella* were independent risk factors for CD (**Table 3**). Note that although *Fusobacterium* was a differential genus with increased abundance in CD, it was not identified as an independent risk factor for CD in the regression analysis.

**Table 3:** Logistic regression analysis of the CD associated genera.

		Univariate analysis			Multivariate analysis		
Phylum	Genus	OR	95%CI	*P*	OR	95%CI	*P*
Bacteroidetes							
	**Prevotella9**	**0.95**	**0.92-0.98**	**0.003**	**0.95**	**0.92-0.98**	**0.003**
	Alistipes	0.901	0.80-1.02	0.099			
	Bacteroides	1	0.98-1.02	0.997			
	Parabacteroides	0.98	0.92-1.05	0.594			
Firmicutes							
	Blautia	0.95	0.89-1.02	0.171			
	Anaerostipes	0.86	0.70-1.05	0.145			
	**Roseburia**	**0.41**	**0.29-0.59**	**0.000002**	**0.4**	**0.28-0.58**	**0.000001**
	**Agathobacter**	**0.54**	**0.35-0.82**	**0.004**	**0.54**	**0.35-0.83**	**0.005**
	**Lachnospira**	**0.24**	**0.12-0.49**	**0.000079**	**0.25**	**0.12-0.50**	**0.000115**
	[Ruminococcus] gnavus group	1.06	0.98-1.15	0.16			
	Lachnoclostridium	1.10	0.94-1.27	0.231			
	**Veillonella**	**1.38**	**1.08-1.75**	**0.009**	**1.5**	**1.10-2.04**	**0.01**
	Megamonas	0.98	0.95-1.01	0.188			
	**Faecalibacterium**	**0.81**	**0.72-0.91**	**0.000335**	**0.82**	**0.73-0.92**	**0.001**
	Phascolarctobacterium	0.96	0.91-1.02	0.156			
	**Romboutsia**	**0.69**	**0.54-0.88**	**0.003**	**0.68**	**0.53-0.87**	**0.002**
	Lactobacillus	1.85	0.81-4.21	0.143			
Proteobacteria							
	**Escherichia-Shigella**	**1.15**	**1.07-1.24**	**0.000112**	**1.15**	**1.07-1.23**	**0.000132**
	**Atlantibacter**	**1.11**	**1.05-1.17**	**0.000246**	**1.11**	**1.05-1.17**	**0.0003**
	Klebsiella	1.03	0.97-1.10	0.343			
	Succinatimonas	NA^a^	NA	0.996			
	Parasutterella	0.92	0.84-1.02	0.121			
Fusobacteria							
	Fusobacterium	1.02	1.00-1.04	0.107			
Actinobacteria							
	Bifidobacterium	0.89	0.77-1.04	0.138			

Adjusted for age and gender in multivariate analysis. ^a^Too few cases.

Bold indicates statistical significance.

### Performance of the microbial markers for CD prediction

Classification models were constructed to evaluate the capability of the 10 differential genera to distinguish CD patients from their healthy relatives. Individually, *Escherichia-Shigella* achieved the highest AUC (0.77) for predicting CD, followed by *Atlantibacter* (AUC = 0.73)*, Veillonella* (AUC = 0.65)*, and Fusobacterium* (AUC = 0.53) (**Figure 4A**). Better prediction power was achieved with an AUC of 0.89 when all 10 abundant differential genera were included in the classification model (**Figure 4B**).

### Correlations between differential genera and biochemical indices

**Figure 5:**
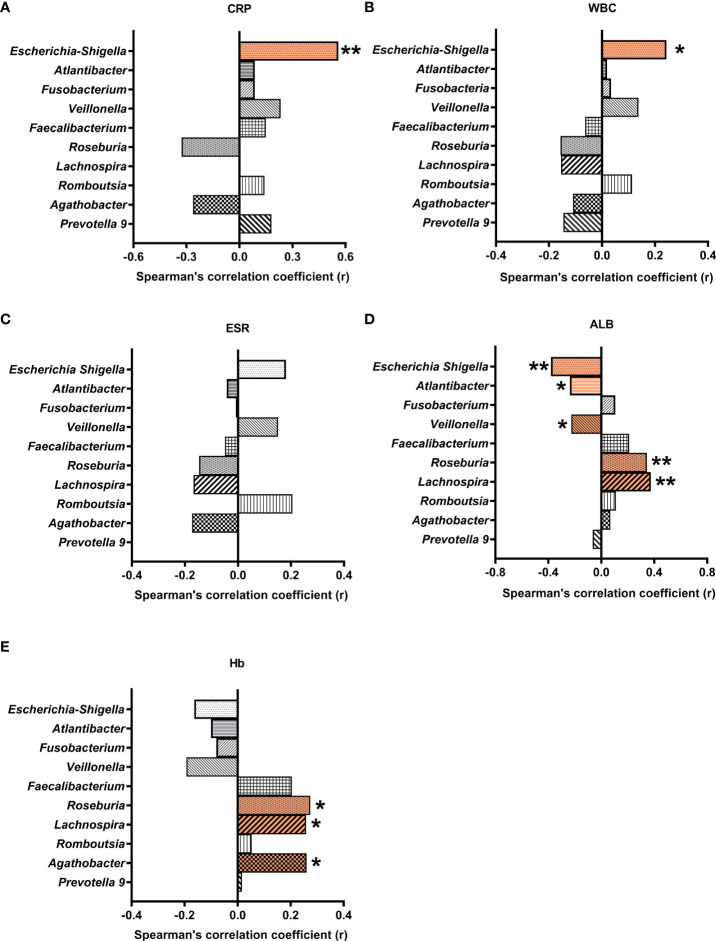
Correlations between differential gut microbial genera and blood biochemical indices. Multiple Spearman’s correlation analyses were conducted with CD and control samples. Correlation coefficients (r) were plotted for microbial genera correlated with CRP **(A)**, WBC **(B)**, ESR **(C)**, ALB **(D)**, and Hb **(E)**. * and ** indicate significant difference at a P value of < 0.05 and < 0.01, respectively. CRP, C-reactive protein; WBC, white blood cell count; ESR, erythrocyte sedimentation rate; ALB, albumin; Hb, hemoglobin.

To understand the possible roles of the gut bacteria in CD pathogenesis, the correlations between the differential genera and relevant biochemical indices were analyzed. We found that both CRP (r = 0.559, P = 0.0009) and white blood cell count (r = 0.243, P = 0.03) were positively correlated with *Escherichia-Shigella* (**Figure 5**). Given the elevated abundance of *Escherichia-Shigella* in CD, our results support a role of *Escherichia-Shigella* in inducing inflammatory markers CRP and white blood cell count.

On the other hand, albumin was negatively correlated with *Veillonella, Escherichia-Shigella, and Atlantibacter*, and positively correlated with *Roseburia* and *Lachnospira* (r = -0225, -0.38, -0.236, 0.344, and 0.374, respectively). Hemoglobin was positively correlated with *Roseburia, Agathobacter*, and *Lachnospira* (r = 0.274, 0.260, and 0.258, respectively).

### Fecal SCFA levels in CD patients compared controls

**Figure 6:**
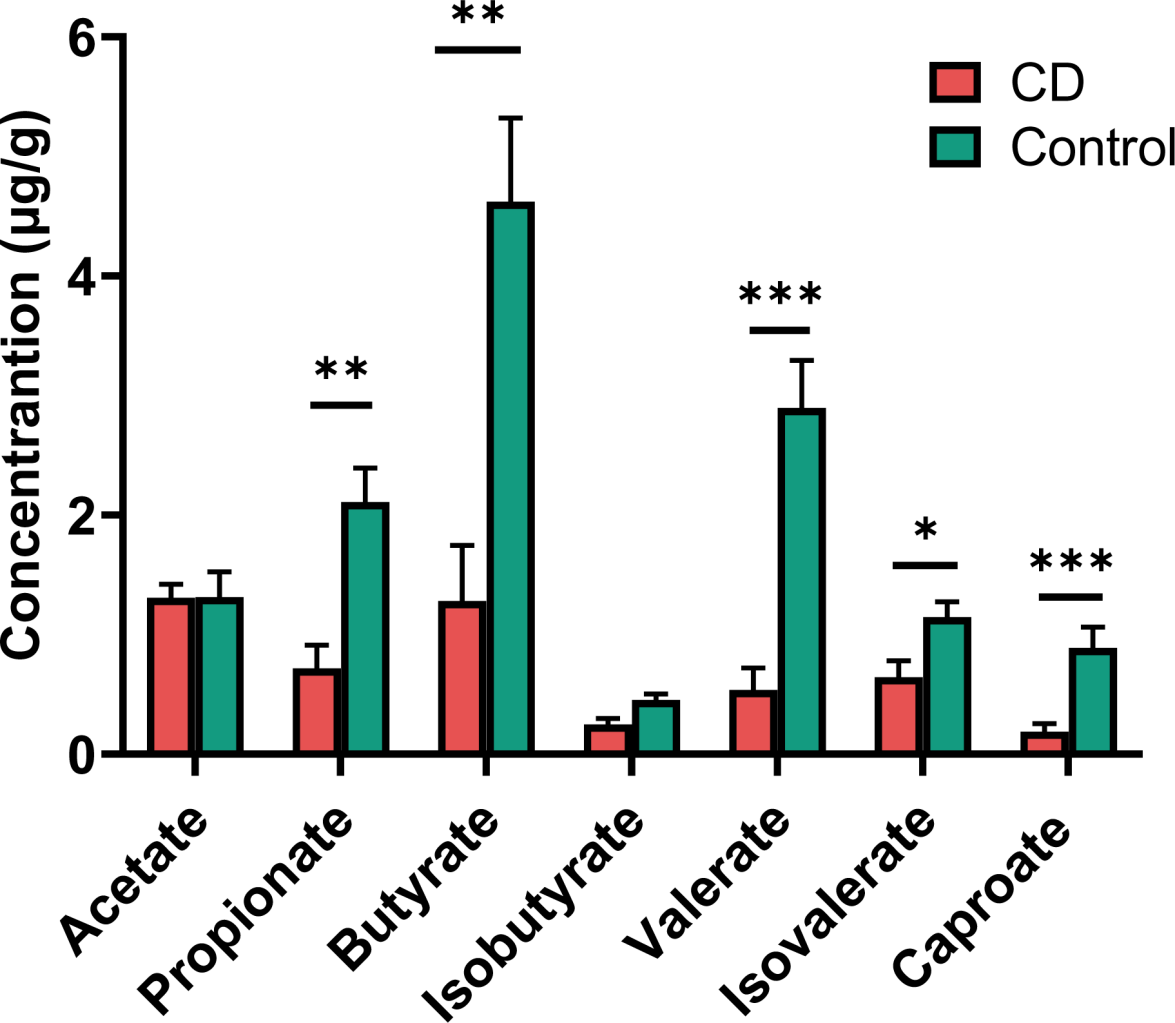
Short chain fatty acids (SCFAs) in the feces of CD patients and their healthy relatives. Propionate, butyrate, valerate, isovalerate and caproate were significantly decreased in CD patients compared to controls. P-values are from two-tailed Student t-tests. *P< 0.05; **P < 0.01; ***P < 0.001.

Many of the differential taxa with decreased abundances in CD are known for SCFA production. Thus the fecal materials were subjected to metabolome analysis targeting SCFAs. CD patients exhibited lower levels of propionate, butyrate, isovalerate, valerate and caproate than their healthy relatives (**Figure 6**). However, similar levels of acetate and isobutyrate were observed between the CD patients and their healthy relatives (**Figure 6**).

### Co-occurrence of *Escherichia-Shigella, Atlantibacter* and SCFA-producing bacteria in the gut of the CD patients and the controls

**Figure 7:**
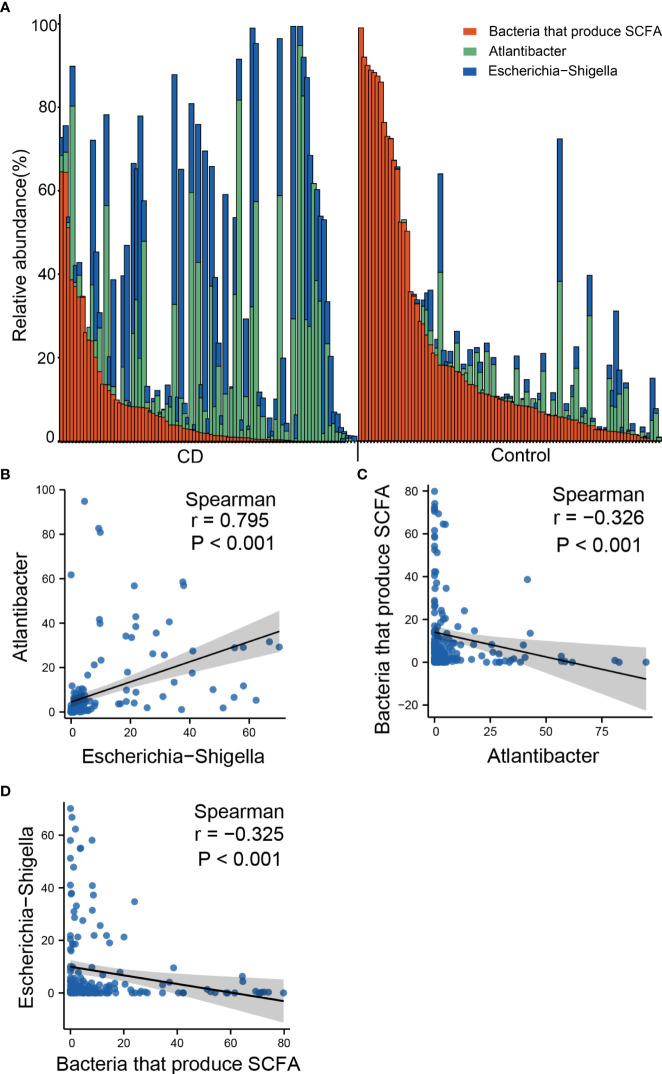
Co-occurrence of altered abundances of Escherichia-Shigella, Atlantibacter and SCFA-producing bacteria. **(A)** Bar plot of relative abundances of Escherichia-Shigella, Atlantibacter and bacteria that produce SCFA. **(B)** Scatter plot showing the correlation of the abundances between *Escherichia-Shigella* and *Atlantibacter*. **(C)** Scatter plot showing the correlation of the abundances between *Escherichia-Shigella* and SCFA-producing genera. **(D)** Scatter plot showing the correlation of the abundances between *Atlantibacter* and SCFA-producing genera. Spearman’s correlation coefficients and *P* values are indicated.

The outstanding alterations observed in the gut of CD include the elevated abundances of *Escherichia-Shigella* and *Atlantibacter*, and decreased abundance of SCFA-producing bacteria. To investigate whether these three types of changes represent independent pathogenic pathways, we examined the co-occurrence of these bacteria in the gut of the CD patients and controls. According to the genus distribution of the CD patients and the controls, *Escherichia-Shigella* and *Atlantibacter* were highly abundant in CD patients, and very often, the elevated abundances of *Escherichia-Shigella* and *Atlantibacter* were accompanied by decreased abundances in SCFA-producing bacteria (**Figure 7A**). In contrast, in most of the healthy controls, *Escherichia-Shigella* and *Atlantibacter* were rare while SCFA-producing bacteria were abundant (**Figure 7A**).

Spearman analyses revealed that the abundance of *Escherichia-Shigella* was positively correlated with that of *Atlantibacter* (**Figure 7B**), and negatively correlated with that of SCFA-producing bacteria (**Figure 7C**). Similarly, the abundance of *Atlantibacter* was correlated with that of *Escherichia-Shigella* and negatively correlated with that of SCFA-producing bacteria (**Figure 7D**). These results indicate that the alterations in these differential genera often occur simultaneously.

### Microbial functional differences between CD patients and healthy relatives

**Figure 8:**
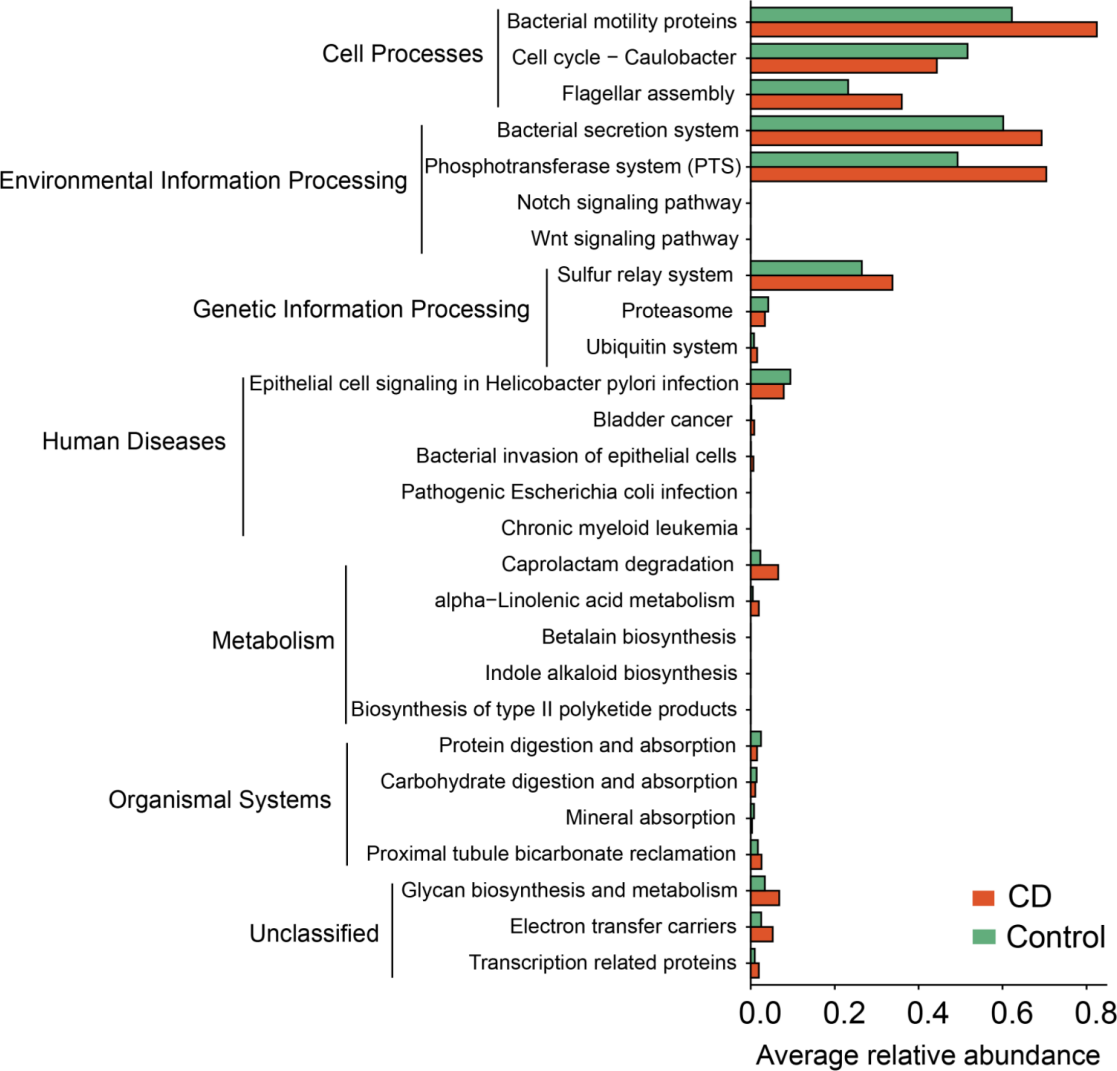
Functional alterations in the microbiota of CD. Based on the estimations with PICRUSt, pathway enrichment analysis was conducted to compare between the CD and the control groups. 27 differential KEGG pathways (*P* < 0.05) in 6 functional categories were identified. *P* values were from paired t-tests.

To understand the functional changes associated with the altered microbial composition in CD, PICRUSt analysis was conducted to estimate the microbial gene functions in the CD and the control groups. 27 functional pathways in 6 functional categories were differentially enriched between the CD patients and their healthy relatives (**Figure 8**). The “pathway”s of bacteria motility proteins, bacteria secretion system, phosphotransferase system, bacteria invasion of epithelial cells, pathogenic *Escherichia coli* infection, caprolactam degradation and glycan biosynthesis and metabolism were enriched in the CD group, while cell cycle-caulobacter, proteasome, protein digestion and absorption, carbohydrate digestion and absorption were enriched in the control group (**Figure 8**).

## Discussion

In this study, the gut microbiota of patients with CD were examined, and compared with the control samples collected from their healthy relatives, so to minimize the impact of genetic and dietary factors on the gut microbiota. Further, we conducted logistic multivariate regression so that the identified microbial markers for CD were less likely influenced by confounding factors common for human studies. Our studies identified 10 abundant genera as differential genera between CD and healthy controls. Among these differential genera, potentially pathogenic *Escherichia-Shigella* and *Atlantibacter* were highly elevated in CD while SCFA-producing *Roseburia, Faecalibacterium*, and *Prevotella 9* were much diminished in the gut of CD. Very often, the reduction of SCFA-producing genera accompanied the elevation of pathogenic genera (*Escherichia-Shigella* and *Atlantibacter*) in the same CD patients, indicating that different pathogenic mechanisms mediated by the over-growth of *Escherichia-Shigella* and *Atlantibacter*, and the reduction of SCFA may simultaneously drive the initiation and development of CD. The differential genera identified in this study demonstrated outstanding capability to serve as diagnosis markers for CD and are potential targets for intervention.

The drastic reduction in microbial diversities reflexes the ecological collapse of the gut microbial communities in CD. Altered representations in CD was observed with many abundant taxa including 3 phyla, 5 families and 10 genera. One frequently observed change is the increased abundance in *Escherichia-Shigella*. Many species in this genus are known to be pathogens or opportunistic pathogens (14, 15). Correlations of *Escherichia-Shigella* with inflammation markers CRP and white blood cell count support a role for *Escherichia-Shigella* in intestinal inflammation. Along this line, the abundance of *Escherichia-Shigella* was inversely correlated with the blood albumin level. Pathogenic *Escherichia-Shigella* species can evade the immune surveillance of the host and induce intestinal inflammation by suppressing epithelial and inflammatory cell autophagy (16).

Functional analysis by PICRUSt supports the role of bacterial infection in CD pathogenesis. Many pathways enriched in CD are known for their involvement in bacterial infection, including “bacterial invasion of epithelial cells”, “pathogenic E. coli infection”, “bacterial motility proteins”, “flagellar assembly”, and “bacterial secretion system”, etc. The functional analysis also identified host inflammation related pathways including “Wnt signaling pathways” and “Notch signaling pathways” (17, 18), supporting a link between bacterial products and intestinal inflammation in CD.

Being members of the same family Enterobacteriaceae, *Atlantibacter* is closely related to *Escherichia-Shigella with >*97% similarity in 16S rRNA gene sequence. *Atlantibacter* showed a similar abundance pattern as that of *Escherichia-Shigella* in the CD patients and the controls. However, unlike *Escherichia-Shigella*, the abundance of *Atlantibacter* was not correlated with the inflammation marker CRP or white blood cell count. Previous studies suggest a role for *Atlantibacter* in bacteremias, urinary tract, and central nervous system infections (19), but there was no report on its possible role in gastrointestinal diseases.

Reduced levels of SCFAs including butyrate and propionate were found in the gut of the CD patients, and this is in line with a reduction of butyrate-producing microbiota including *Roseburia* and *Faecalibacterium*. CD microbiota also exhibited reduced abundance in *Prevotella*, which produces acetate (20). However, no difference in serum acetate level was observed between CD and control groups, which may be explained by reduced consumption of acetate for butyrate production (21) in CD microbiota. As the major energy source for enterocyte, butyrate help to maintain the intestinal barrier function (22). In addition, butyrate and propionate may activate regulatory T cell function (23), and reduce neutrophil recruitment through blockade of IL8 production (24). Thus, reduced levels of SCFA-producing bacteria, and consequently reduced levels of SCFAs, may contribute to intestinal inflammation in CD.

Therefore, two mechanisms were implicated to explain the contributions of the altered gut microbiome in CD pathogenesis: overgrowth of pathogenic *Escherichia-Shigella*, and reduction of SCFA-producing bacteria. Our data indicated that these two mechanisms often co-exist in the same patient, as elevated abundance of *Escherichia-Shigella* is usually accompanied by reduced SCFA-producing bacteria. Since *Escherichia-Shigella spps.* are adhesive bacteria forming biofilms that colonize the mucosal surface (25, 26), it is possible that the reduction in SCFA-producing bacteria is a consequence of increased colonization of adherent *Escherichia-Shigella spps.* Possible causes for increased abundance in *Escherichia-Shigella spps.* include high fat diet and abnormality in fucosylation of the mucosal proteins (6).

In summary, the gut microbiome of CD was examined using healthy relatives as controls so to minimize the impact of genetic and environmental influences. In addition, logistic multivariate regression was conducted to further reduce the influence of confounding factors. This way, the obtained differential genera are correlated with blood inflammatory markers, and exhibited a high capacity to distinguish between CD and healthy controls. Our data suggest that elevated opportunistic pathogen *Escherichia-Shigella* and reduced SCFA-producing bacteria likely mediate two pathomechanisms of CD and these two mechanisms often co-exist in the same patient. The differential bacteria we identified may serve as diagnosis markers for CD and are potential targets for intervention.

## Materials and methods

### Participant

Fecal samples of 91 CD patients and 91 their healthy relatives were collected at the Sixth Affiliated Hospital of Sun Yat-sen University in Guangzhou, China, between March 2014 and December 2019. Patients with CD were diagnosed on the basis of standard clinical, endoscopic, and histological criteria. To diagnose CD, intestinal infections were ruled out by stool routine test, stool culture, and *Clostridium difficile* testing. Patients with an available healthy relative were enrolled. Exclusion criteria included any prior history of digestive tract-related diseases or surgeries, other than CD, such as gastrointestinal polyp, intestinal adenoma, gastrointestinal tumors; the use of antibiotics or proton pump inhibitors in the past month. This study was approved by the Institutional Review Board of the Sixth Affiliated Hospital, Sun Yat-sen University. The ethics approval number is 2014ZSLYEC-003. Informed consent was obtained from all participants.

### Sample collection and DNA isolation

Fresh fecal samples were collected in a sterile container, and immediately stored at -80°C. Microbial genomic DNA was isolated with a stool DNA Kit (OMEGA; cat. #D4015-01) from fecal samples according to manufacturer’s instruction. The total DNA was stored at -80°C until used for PCR.

### Microbiome analysis

DNA was sequenced at BGI (Shenzhen, China). The sequencing of V5-V6 region of 16S rRNA gene was performed with a paired-end method using the Illumina MiSeq Benchtop Sequencer. Sequencing reads were analyzed with the Quantitative Insights Into Microbial Ecology 2 (QIIME2) version 2019.7. The FASTQ files were used to perform quality control on the raw sequences. “demux emp-paired” method of q2-demux plugin was used to demultiplex sequencing reads followed by quality filtering and denoising with “dada2 denoise-paired” method using q2-dada2 plugin available at QIIME2, which generates the table of amplicon sequence variants (ASVs). The 16S rRNA sequencing data are available at the Bio-Med Big Data Center (https://www.biosino.org/bmdc/, project ID: OEP002161). The core-metrics-phylogenetic method was used to analyze α and β diversities. The significant differences in α diversity were calculated using the α-group-significance command in QIIME2. Principle coordinate analysis (PCoA) plots were created with MicrobiomeAnalyst. The statistical significance of the separation among groups was assessed by the linear discriminant analysis (LDA) effect size (LEfSe) method based on linear discriminant analysis scores established by Curtis Huttenhower (http://huttenhower.sph.harvard.edu/galaxy/), using the criteria of P <0.05 and LDA score >3.5. Logistic multivariate regression analysis was performed to identify potential associations between individual bacteria genera and disease status (CD or healthy). In addition to the microbial marker identified in the univariate analysis, the influence of age and gender were considered in this binary multivariate regression analysis.

### Functional predictions

Functional prediction of 16S rRNA was conducted using Phylogenetic Investigation of Communities by Reconstruction of Unobserved States (PICRUSt) to normalize ASVs (27). Normalized ASVs were then categorized into Kyoto Encyclopedia of Genes and Genome (KEGG) Orthologg (KO) in the greengene ID database. The descriptive and related functional information of each KO, including KO, pathway, and enzyme information, were obtained from the KEGG database, and the abundance of each functional category was calculated.

### Short chain fatty acids targeted metabolomics analysis

Concentrations of the short-chain fatty acids in intestinal contents were measured at the BGI (Shenzhen, China) using gas chromatography–mass spectrometry (GC-MS). Quantities of metabolites in fecal samples were analyzed using standard calibration curves. Method validation was conducted by evaluating the correlation (R^2^ > 0.99) of the linearity, accuracy and repeatability.

### Additional statistical methods

Descriptive data are expressed as mean ± SEM or count with percentage as appropriate. Paired-t tests were performed to evaluate differences in taxonomic abundance between the study groups. Results with p-value <0.05 were considered significantly different. Correlation analysis was performed with Spearman’s test using Statistical Package for the Social Sciences (SPSS, version 26.0).

## Data availability statement

The datasets presented in this study can be found in online repositories. The names of the repository/repositories and accession number(s) can be found below: https://www.biosino.org/bmdc/, OEP002161.

## Ethics statement

The studies involving human participants were reviewed and approved by Institutional Review Board of the Sixth Affiliated Hospital, Sun Yat-sen University. The patients/participants provided their written informed consent to participate in this study.

## Author contributions

LZ and MZ conceived and designed this study. JY, JH, XL, JW, YZ and MZ collected patient samples and clinical data. JH, SC, YL and WW performed experiments. JH, SC, JY, WW, LZ and MZ analyzed data. SC, JH and LZ prepared the manuscript. All authors critically revised the manuscript. All authors had access to the study data and had reviewed and approved the final manuscript.

## Funding

This work was supported by the Sun Yat-sen University Clinical Research 5010 Program 2014008(MZ), Guangdong Province “Pearl River Talent Plan” Innovation and Entrepreneurship Team Project 2019ZT08Y464 (LZ), the National Natural Science Foundation of China 81900490 (JY), 81770571 (LZ), 91942303(MZ), and 81670477(MZ), and the program of Guangdong Provincial Clinical Research Center for Digestive Diseases (2020B1111170004).

## Conflict of interest

The authors declare that the research was conducted in the absence of any commercial or financial relationships that could be construed as a potential conflict of interest.

## Publisher’s note

All claims expressed in this article are solely those of the authors and do not necessarily represent those of their affiliated organizations, or those of the publisher, the editors and the reviewers. Any product that may be evaluated in this article, or claim that may be made by its manufacturer, is not guaranteed or endorsed by the publisher.

## Abbreviations

ASV, amplicon sequence variant; AUC, area under curve; BMI, body mass index; CD, Crohn’s disease; CRP, C-reactive protein; ESR, erythrocyte sedimentation rate; KEGG, Kyoto Encyclopedia of Genes and Genome; KO, Kyoto Encyclopedia of Genes and Genome (KEGG) Orthology; LDA, linear discriminant analysis; LEfSe, linear discriminant analysis effect size; PCoA, principle coordinate analysis; PICRUSt, Phylogenetic Investigation of Communities by Reconstruction of Unobserved States; QIIME2, quantitative Insights Into Microbial Ecology 2; SCFA, short chain fatty acid.
